# Hydrogen separation through tailored dual phase membranes with nominal composition BaCe_0.8_Eu_0.2_O_3-δ_:Ce_0.8_Y_0.2_O_2-δ_ at intermediate temperatures

**DOI:** 10.1038/srep34773

**Published:** 2016-11-04

**Authors:** Mariya E. Ivanova, Sonia Escolástico, Maria Balaguer, Justinas Palisaitis, Yoo Jung Sohn, Wilhelm A. Meulenberg, Olivier Guillon, Joachim Mayer, Jose M. Serra

**Affiliations:** 1 Institute of Energy and Climate Research IEK-1, Forschungszentrum Jülich GmbH, D-52425 Jülich, Germany; 2 Instituto de Tecnología Química, Universidad Politécnica de Valencia-Consejo Superior de Investigaciones Científicas, Av. Naranjos s/n, E-46022 Valencia, Spain; 3 Ernst Ruska-Centre for Microscopy and Spectroscopy with Electrons ER-C, Forschungszentrum Jülich GmbH, D-52425 Jülich and Central Facility for Electron Microscopy GFE, RWTH Aachen University, 52074 Aachen, Germany

## Abstract

Hydrogen permeation membranes are a key element in improving the energy conversion efficiency and decreasing the greenhouse gas emissions from energy generation. The scientific community faces the challenge of identifying and optimizing stable and effective ceramic materials for H_2_ separation membranes at elevated temperature (400–800 °C) for industrial separations and intensified catalytic reactors. As such, composite materials with nominal composition BaCe_0.8_Eu_0.2_O_3-δ_:Ce_0.8_Y_0.2_O_2-δ_ revealed unprecedented H_2_ permeation levels of 0.4 to 0.61 mL·min^−1^·cm^−2^ at 700 °C measured on 500 μm-thick-specimen. A detailed structural and phase study revealed single phase perovskite and fluorite starting materials synthesized via the conventional ceramic route. Strong tendency of Eu to migrate from the perovskite to the fluorite phase was observed at sintering temperature, leading to significant Eu depletion of the proton conducing BaCe_0.8_Eu_0.2_O_3-δ_ phase. Composite microstructure was examined prior and after a variety of functional tests, including electrical conductivity, H_2_-permeation and stability in CO_2_ containing atmospheres at elevated temperatures, revealing stable material without morphological and structural changes, with segregation-free interfaces and no further diffusive effects between the constituting phases. In this context, dual phase material based on BaCe_0.8_Eu_0.2_O_3-δ_:Ce_0.8_Y_0.2_O_2-δ_ represents a very promising candidate for H_2_ separating membrane in energy- and environmentally-related applications.

Hydrogen permeation membranes are a key element to reach high energy conversion efficiency and decreasing the greenhouse gas emissions from power generation and energy-intensive industries, i.e. by capturing and utilizing CO_2_ or moving towards hydrogen-based systems by extracting highly pure H_2_ from gas mixtures^1^,^2^,^3^. In this context, the integration of high performance H_2_-permeation ceramic membranes with competitive manufacturing cost and life time (>3 years) in industrial processes can significantly intensify them by (i) maximizing products yield and energy efficiency and (ii) promoting the reaction rates^4^. Such integrative membrane approach of *in situ* H_2_ extraction (used as fuel) or consumption (as raw material for chemical production) in a proton conductor-based membrane reactor would shift the thermodynamic equilibrium towards the product side, hence boosting process efficiency, saving energy and reducing final product cost- all factors of high environmental and economic impact. Proton conducting ceramic membranes may therefore be of interest for integrated gasification process^5^,^6^, and more specifically in water gas shift reactors at elevated temperatures (600–900 °C)^7^, in catalytic membrane reactors (CMRs) for accomplishing chemical and petro-chemical reactions (e.g. ammonia synthesis^8^,^9^, non-oxidative de-hydrogenation reaction^10^).

Although every specific application field sets particular requirements in terms of membrane stability, as a general rule, a good ceramic candidate has to remain phase and chemically stable at elevated temperatures in atmospheres possibly containing H_2_, H_2_O, CO, CO_2_, CH_4_, SO_x_, H_2_S, and different levels of ash. Such atmospheres represent extreme operating environments for the majority of H_2_ selective material classes, leading to material decomposition, membrane disintegration and performance degradation. Specifically, formation of undesired phases (carbonates, hydrates, sulfates) is critical for ceramic based membranes, while hydrogen-induced embrittlement and sulfur poisoning are particularly critical for Pd-based membranes^7^,^11^.

Properties like high selectivity for H_2_, significant durability, mechanical and hydrothermal stability in reducing environments, as well as operating temperature ranges above 500 °C, make dense ceramic mixed proton-electronic conductors a promising alternative to precious metals and related alloys (Ag-Pd)^12^, polymers^13^, metal-organic frameworks (MOFs)^14^,^15^, zeolite^16^ or other ceramic microporous materials^17^,^18^.

Apart from the stability issues which emerge to be solved, performance target for H_2_-flux of 1–2 mL_n_·min^−1^·cm^−2^ at 600–700 °C has been set for H_2_-separation dense ceramic materials (based on the technical targets for dense metallic membranes for H_2_ separation and purification). However, these values have not been yet achieved.

In the context of above, scientific community still faces the challenge of identifying highly performing and at the same time stable mixed proton-electronic conducting ceramic materials and to explore and utilize them as membranes in a number of chemical reactions and H_2_ separation tasks at elevated temperatures.

In search for promising candidates for H_2_ separation applications, electrical and proton transport properties of large number of oxide ceramics from several structural classes have been explored with particular intensity in the last years. Hence, a wide range of state-of-the art perovskite-based proton conductors^19^,^20^ to materials with less trivial structures and relatively novel to H_2_ extraction application, as defective fluorites^21^,^22^,^23^,^24^,^25^,^26^, pyrochlores^27^, fergusonites^28^,^29^,^30^,^31^,^32^ has been covered. A comprehensive review on the electrical properties of a number of proton conductors could be found in ref. 33, while Table 1 presents a comparison of thickness-normalized H_2_ permeation flux values from literature. As it could be inferred from the table, H_2_-permeation rates still remain lower than the milestone value at the target temperature.

The largest number of studies is however dedicated to the perovskite-based oxide ceramic materials, in particular with large lattice constant of the type SrCeO_3_, BaCeO_3_ and BaZrO_3_ (Table 1). Substituted BaCeO_3_ exhibits the highest levels of proton thermodynamic stability in the crystal structure remaining fully hydrated up to temperatures of about 600 °C but it is unstable in acidic gas environments due to its alkaline character^19^,^66^,^67^,^68^.

Apart from their good proton conduction, perovskite-based materials e.g. of the type BaCe_1-x_M_x_O_3-δ_ exhibit also electronic conduction upon doping/substitution with M-cations with mixed valence^40^,^42^,^69^. However, the levels of electronic conduction are significantly low relative to the ionic conduction. Indeed, a raise in the level of electronic conductivity can be pursued by adding a second electronically conducting crystalline phase to the pristine proton conductor. As reported by Elangovan *et al*.^61^, a successful approach to obtain H_2_-permeating material with high performance, is to combine (i) a protonic conducting perovskite phase BaCe_1-x_M_x_O_3-δ_ and (ii) an electronic conducting fluorite phase Ce_1-y_M_y_O_2-δ_ (M is metal dopant) into the so called *dual-phase ceramic material*, which at the end possesses increased ambipolar conductivity. The two crystalline phases form the *cer-cer composite* with certain degree of percolation, which provides efficient pathways for proton and electronic transport across the membrane. They are furthermore in a close contact at high temperature and reducing environments, therefore sufficient chemical and thermal compatibility between them is required. Elangovan *et al*.^61^, Ricote *et al*.^70^, Medvedev *et al*.^71^ and Rebollo *et al*.^63^ have shown the chemical compatibility and stability of systems consisting of BaCe_1-x_Ln_x_O_3-δ_:Ce_1-x_Ln_x_O_2-δ_ (x = 0.1–0.2) and BaCe_0.65_Zr_0.2_Y_0.15_O_3-δ_:Ce_0.85_Ln_0.15_O_2-δ_ (Ln = Y, Gd), while Huang *et al*.^72^ reported the appearance of minor additional phase in the grain boundaries when Zr is doped in the perovskite phase. Furthermore, the addition of the doped ceria phase would enhance the stability of otherwise easily attacked by CO_2_ and H_2_O-containing environments BaCeO_3_ phase due to a shift in thermodynamic equilibrium towards the reactant side^73^. This leads to overall suppression of BaCeO_3_ decomposition to BaCO_3_, Ba(OH)_2_ and CeO_2_.

In the present work composite ceramic membrane material with nominal composition BaCe_0.8_Eu_0.2_O_3-δ_:Ce_0.8_Y_0.2_O_2-δ_ (labeled BCEO:CYO) at 50:50 vol.% ratio was explored as H_2_ permeation membrane in the temperature range 600–700 °C. This ratio was selected to ensure the largest degree of interaction between the two phases and sufficient transport pathways both for protons and electrons. In the context of above, acceptor substitution with 20 mol. % Eu^3+^ in BaCeO_3_ and the same amount of Y^3+^ in CeO_2_ was undertaken as a strategy to pursue an improvement and fine-tuning of transport properties of the composite ceramic material.

For the selection of the two substituents several factors were accounted. BaCeO_3_ doped by Gd from rare earths (RE) in addition to Y, exhibits the highest ionic (protonic) conductivity among solid solutions BaCe_1-x_RE_x_O_3-δ_^74^,^75^,^76^. Eu^3+^ (4f^6^) and Gd^3+^ (4f^7^) have the same valence state, nearly the same ionic radii and good geometrical compatibility with Ce as B-site ion in Ba-cerate. The ionic radius increases in the row Gd→Eu→Sm and decreased protonic conductivity has been found for Sm due to the increased lattice distortion. However, similar lattice distortions and conductivities are expected to be produced by Eu^3+^ and Gd^3+^ in BaCeO_3_. As demonstrated elsewhere^77^, slightly higher values of the total conductivity in H_2_ environments were determined for BaCe_0.85_Gd_0.15_O_3-δ_ in comparison with BaCe_0.85_Eu_0.15_O_3-δ_. Furthermore, there was practically no difference in conductivities under H_2_/N_2_ and air atmospheres measured for BaCe_0.85_Eu_0.15_O_3-δ_, while for BaCe_0.85_Tb_0.15_O_3-δ_ largely higher values were recorded under reducing environments. According to Radojkovic^78^, Eu^3+^ substitution in BaCeO_3_ leads to clear advantages over that with Y^3+^ or Gd^3+^ reported as the best proton conductors. One may highlight (i) the lower sintering temperature (reported to be below 1500 °C); (ii) the larger conductivity under wet H_2_ conditions (1.21·10^−2^ S·cm^−1^ at 600 °C) due to the larger unit cell volume and lattice distortion favoring intra-octahedral proton migration of lower activation energy; (iii) improved grain boundary conductivity ascribed to a decrease in band gap energy related to the transition between O *2p* valence band to Ce *4f* conduction band; and (iv) dominating proton conductivity under OCV conditions at 650 °C and 700 °C.

On the other hand, in reducing atmospheres, Y-substituted CeO_2_ exhibits predominantly electronic conduction via small polaron hopping mechanism due to Ce^4+/3+^ variable valence. Besides, detailed structural and phase characterization via Rietveld refinement on XRD will help to evaluate the phase formation and phase stability of the cer-cer composite supported by microstructural study via SEM and HR-TEM. Electrical conductivity properties of the composite material and its constituting phases were studied to elucidate the relative contributions of each phase in the composite. In addition, hydrogen permeation of the composite membrane with thickness of 500 μm was characterized as a function of the temperature, the hydration conditions and the hydrogen concentration in the feed. Membrane stability in CO_2_-containing atmospheres was studied *in situ* with H_2_ permeation measurements to conclude on eventual phase, microstructural and performance degradation effects.

## Results and Discussion

### Structural and phase composition study

**Figure 1:**
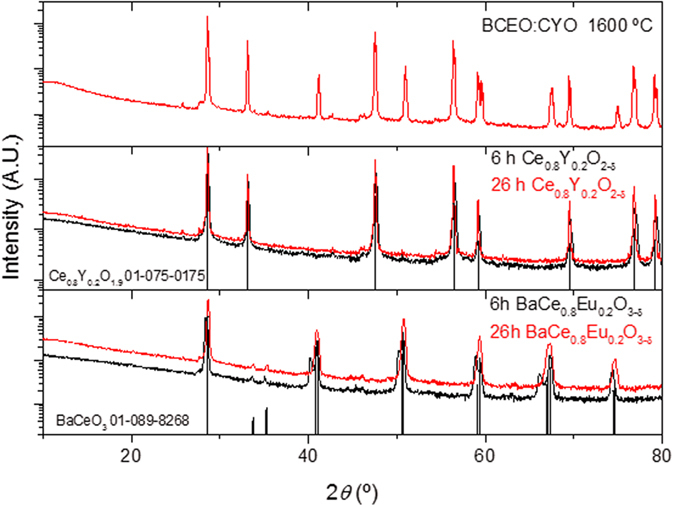
XRD diffraction patterns of BCEO (bottom); CYO (middle) synthesized by solid-state reaction at 1400 °C for 6 and 26 h; and the BCEO:CYO (top) composite pattern sintered for 10 h at 1600 °C.

Figure 1 shows the XRD patterns of BCEO and CYO synthesized via the conventional solid**-**state route at 1400 °C, and the composite pattern resulting from mixing them in a 50:50 vol.% ratio and sinter for 10 h at 1600 °C. Rietveld refinement on the XRD performed on the BCEO and CYO separately showed that the single phases were not formed after 6 h of calcination. XRD patterns revealed a mixture of tetragonal and orthorhombic perovskite phases for BCEO and two fluorite phases with different Y content (details are summarized in the Table 2). An extra thermal treatment of 20 h (in total 26 h) allows single phase samples with sharp defined peaks to be obtained. Besides, once the pressed pellet is sintered at 1600 °C, the observed splitting of peaks disappear and XRD reveals that a single BCEO perovskite phase and a single CYO fluorite were formed. The presence of other oxides was not observed.

As it can be observed from Table 2, the lattice parameters of the two phases constituting the dual phase ceramics disclosed certain deviation from the nominal stoichiometry. The ceria phase shows a cubic fluorite structure with a lattice parameter of 5.411 (1) Å, clearly shifted from the theoretical value of 5.405 Å estimated by Kim’s empirical formula^79^ for 20 mol.% yttria substitution in CeO_2_ (and from the 5.405 Å obtained experimentally for the single doped ceria phase). The calculation took into account the ionic radii in 8-fold coordinated Ce^4+^ and Y^3+^ cations, which are 0.97 and 1.019 Å, respectively. Since the ionic radii of Eu^3+^ is 1.066 Å, the substitution of Ce^4+^ cations by Eu^3+^ is suggested. By using the experimental cell parameter obtained for the fluorite phase, Kim’s formula can be reversely used to estimate to which extent Eu migrated to the ceria phase. As there are not yttrium related impurities, the yttrium content in the fluorite was fixed to 20 mol.%, which gives a value of x = 10.5 mol.% for Eu^3+^ and corresponds to stoichiometry Ce_0.695_Y_0.2_Eu_0.105_O_2-δ_. This value indicates that practically all the Eu introduced by the perovskite phase has migrated to the fluorite phase. The mass balance by ICP-OES (Supplementary information, Table S1) indicates that the barium was not deficient after the sintering (see Experimental Section, Sample preparation paragraph). The unit cell volumes of pure BaCeO_3_, BaCe_0.9_Eu_0.1_O_2.95_ according to^78^, BaCe_0.8_Eu_0.2_O_2.9_ after 26 h at 1400 °C and for Ba-cerate phase in the composite material BaCe_0.8_Eu_0.2_O_2.9_:Ce_0.8_Y_0.2_O_1.9_ after 10h at 1600 °C are respectively: 340 Å^3^, 341.79 Å^3^, 342.4 Å^3^ and 340.73 Å^3^, showing that (i) with increasing the Eu amount the volume expands as expected; and (ii) Ba-cerate in the composite is practically Eu-depleted.

### Microstructural characterization

**Figure 2:**
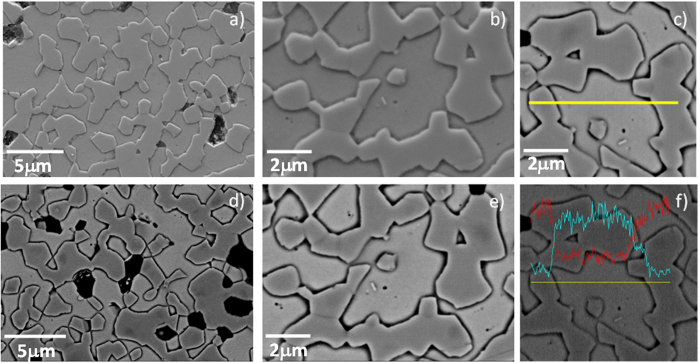
(**a**,**b**) SEM, (**c**–**e**) BSE-SEM and (**f**) EDS linescan analysis of the fractured cross-section of the BCEO:CYO membrane sintered at 1600 °C.

FE-SEM analysis of the polished cross-section of the BCEO:CYO membrane revealed a high relative density (>98%), although some occluded porosity is still present (Fig. 2a–f). The thermal expansion coefficient (TEC) of the composite material in air (12.4·10^−6^ K^−1^) shows identical behavior during heating and cooling cycles in air (Supplementary information, Figure S2). No cracks related to the TEC thermochemical cycles were detected due to the good thermal compatibility of the two ceramic components. BSE-SEM (Fig. 2c–e) and EDS analysis (Fig. 2f) show dark grains corresponding to the CYO phase, as well as a bright phase corresponding to BCEO. In addition, Fig. 2c,f shows the EDS linescan analysis of a fracture cross-section of the membrane and the corresponding Ba and Y concentration profiles. Neither Ba (in Fig. 2f the blue line) nor Y (in Fig. 2f the red line) interdiffusion between both phases occurs. The topography and distinct textures observed in the polished cross-section for each phase originates from the difference in hardness, i.e., the dark phase (CeO_2_) is harder, and this effect was previously reported for BaCeO_3_:CeO_2_ composites^70^.

**Figure 3:**
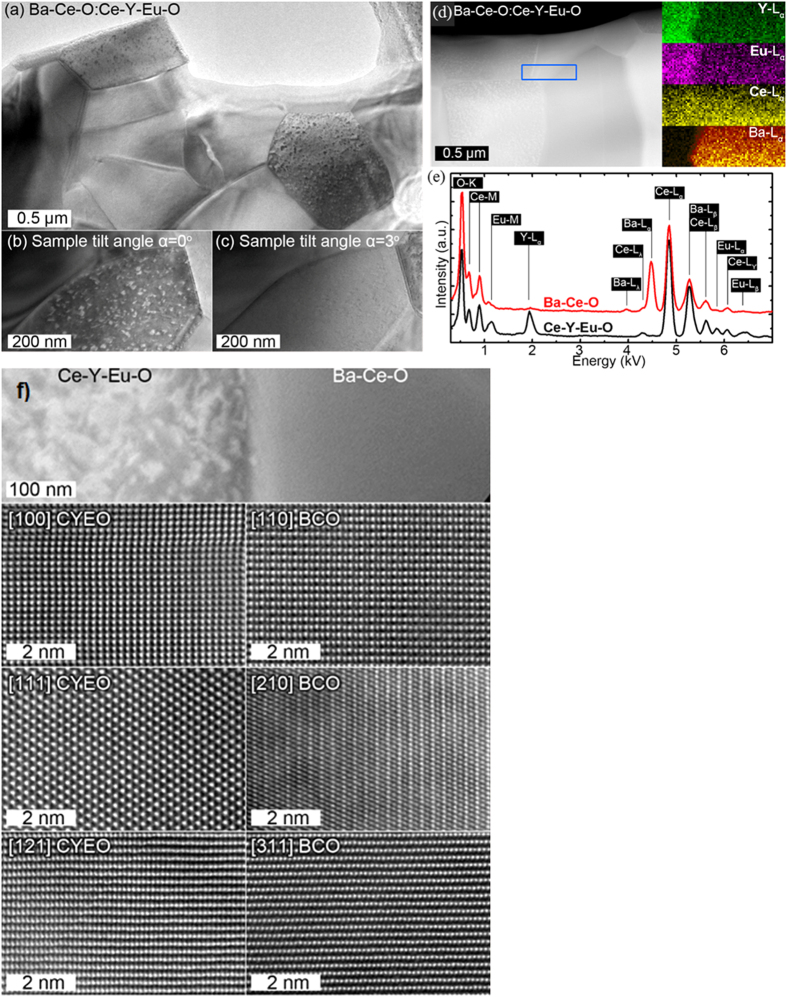
(**a**) Overview TEM image acquired from BCEO:CYO sample sintered at 1600 °C for 10 h; (**b**)-(**c**) shows the grain contrast dependence with distinct sample orientation (tilt); (**d**) Overview STEM-HAADF acquired from BCEO:CYO samples together with corresponding elemental EDX areal maps; (**e**) integrated EDX spectrum from the CYO and BCEO grains; and (**f**) High-resolution STEM-HAADF images acquired from CYO (Ce-Y-Eu-O) grain (left column) and BCEO (Ba-Ce-O) grain (right column) in the BCEO:CYO dual phase sample utilizing different crystallographic projections.

Further detailed insight in composite microstructure was achieved by the TEM and high resolution scanning transmission electron microscope with high-angle annular dark-field imaging (HRSTEM-HAADF) (Fig. 3). A low-magnification TEM image acquired from the composite sample as-sintered at 1600 ^o^C is shown in Fig. 3a (Composite sample, nominally labeled BCEO:CYO, was actually named BaCeO:CeYEuO in the TEM image after its actual composition with Eu predominantly located in the CYO phase). The sample is composed of randomly oriented, well-connected and packed grains with sizes ranging from 0.5 μm to 2 μm. The grains have a polygonal shape, which minimizes their surface energy. Two types of grains were observed: some grain’s interiors were decorated with dotty/fuzzy contrast, while other grains surface was even. Furthermore, dotty contrast inside grains is sensitive to the grains relative orientation with respect to the electron beam. The slight tilt of the grain could enhance or diminish the dotty contrast significantly (Fig. 3b,c). HR-TEM investigation does not reveal any interface phases at the grain boundaries (not shown). STEM–HAADF images acquired using strong elemental contrast (Z-contrast) conditions are shown in Fig. 3d. The dotty/fuzzy contrast is evidenced under such imaging conditions as well. EDX analysis (Fig. 3d,e) revealed that dotty/fuzzy contrast rich grains are composed of Ce, Y, Eu and O elements, while other grains consist of Ba, Ce and O. The dotty pattern contrast might be attributed to Eu presence in CYO grains, which is associated with high point-defect density caused by incorporation of Eu into this phase. Furthermore, no migration of Y from the fluorite to the perovskite could be evidenced (Fig. 3(e)). HR-STEM-HAADF images recorded from the two grain types in range of crystallographic projections exhibit single crystal structure as shown in Fig. 3f. Dotty contrast is absent in HRSTEM-HAADF imaged for CYO (Ce-Y-Eu-O) grain due to channeling contrast mechanisms dominant at those imaging conditions. By correlating *s*elected area electron diffraction (SAED) and HRSTEM-HAADF images with crystal structure data, the CYO (actually CYEuO) grains showed Fm-3m space group, while BCEO (actually BCO) grains exhibit Pmcn space group crystal structure, both in agreement with the Rietveld refinement made on the XRD patterns. As a summary, the microstructural analysis confirms that Eu diffused from the BCEO perovskite to the CYO fluorite when exposed to high temperature, as previously shown in the XRD section with no evidence for Y diffusion from the fluorite to the perovskite.

Since the composition and microstructure of both phases is pure, without grain boundary segregations electrical and electrochemical study was performed in order to evaluate the transport properties of this dual-phase material.

### Electrochemical characterization

**Figure 4:**
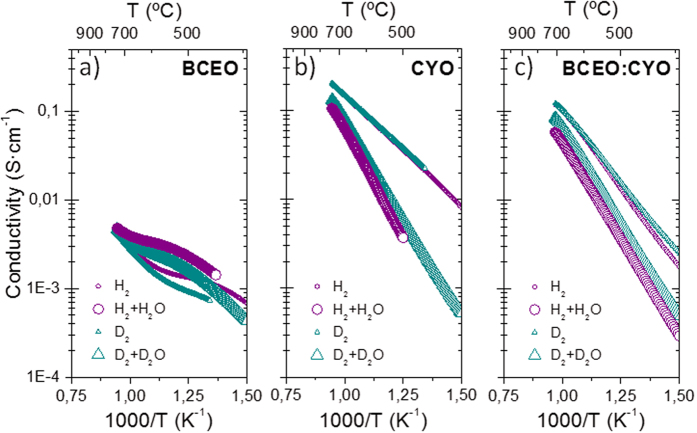
Total conductivity as a function of inverse temperature measured under four different reducing atmospheres: 5 vol.% H_2_ in He and 5 vol.% D_2_ in He both dry and moist (H_2_ and D_2_ humidified with H_2_O and D_2_O at room temperature, respectively) for (**a**) BCEO, (**b**) CYO and (**c**) BCEO:CYO composite.

Figure 4 displays the total conductivity corresponding to the single phase materials BaCe_0.8_Eu_0.2_O_3-δ_ (BCEO) and Ce_0.8_Y_0.2_O_2-δ_ (CYO), and the composite material BCEO:CYO with nominal composition BaCe_0.8_Eu_0.2_O_3-δ_:Ce_0.8_Y_0.2_O_2-δ_. The samples have been measured as a function of inverse temperature under H_2_, H_2_+H_2_O, D_2_ and D_2_+D_2_O atmospheres. Within the studied range of temperatures (from 800 °C to 400 °C), BCEO exhibits mainly protonic conductivity, ascertained from the H/D isotopic and hydration effect, i.e. lower conductivity in D_2_ than in H_2_ atmospheres and higher conductivity in wet than in dry atmospheres. Above 750 °C, the different conductivity curves converge due to the progressive oxide dehydration with temperature, leading to significant drop in proton concentration. On the other hand, CYO possesses higher conductivity in dry atmospheres than in wet conditions (wet atmospheres signify less reducing conditions than dry atmospheres) suggesting that electronic transport prevails under these conditions. Finally, BCEO:CYO composite shows prevailing *n*-type conduction behaviour and conductivity values similar to CYO, indicating proper percolation of this electron conducting component, as previously suggested by the SEM analysis.

**Figure 5:**
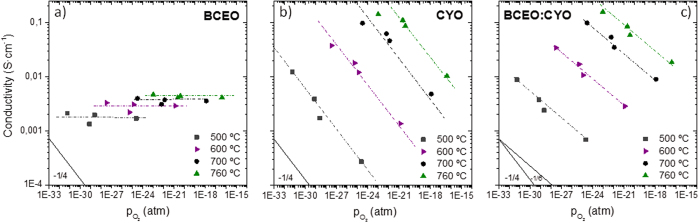
Total conductivity as a function of *p*O_2_ under wet reducing atmospheres at four different temperatures for (**a**) BCEO, (**b**) CYO and (**c**) BCEO:CYO.

In order to disclose the predominant transport of these compounds, *p*O_2_ effect on the conductivity was also studied under wet reducing conditions (Fig. 5). The BCEO behavior as a prevailing proton conductor deduced from the observed H/D isotopic effect is confirmed by the relationship σ∝*p*O_2_^0^ in all studied temperatures i.e., the conductivity is independent on the *p*O_2_ values. On the other hand, CYO exhibits predominant *n*-type electronic conduction following a power law of σ∝*p*O_2_^−1/4^ as it has been reported previously for CYO material^80^,^81^. Finally, from the relationship σ∝*p*O_2_^−1/6^ observed for the composite BCEO:CYO, it can be assumed an intermediate behaviour, i.e. it is mainly *n*-type electronic conductor but presents a significant ionic contribution, arising from the physical mixture of the two different conductors.

Temperature programmed reduction (TPR) measurements (Figure S3 in Supplementary Information) were performed in order to study the redox behavior of the single compounds and the composite, and to correlate with the conductivity data. The CYO sample shows the maximum of its reduction peak at 725 °C, ascribed to the reducible bulk Ce^4+^. This reduction temperature agrees with previous studies on doped CeO_2_^82^. Regarding BCEO, a broad reduction peak is observed between 300–700 °C, which can be attributed to the Ce^4+^ reduction and some oxygen release. The reduction of Eu^3+^ to Eu^2+^ is not expected in the considered temperature range as it was observed previously for Eu doped NdWO compound^83^. Finally, the Ce^4+^ reduction in the sintered BCEO:CYO samples is shifted to higher temperatures presenting its maximum at 800 °C. The lower reducibility of Ce^4+^ in this sample could be ascribed to the different stoichiometry of the BCEO and doped ceria grains as compared with the separate single material, as this is previously stated for instance in heavily doped cerias^84^.

### Hydrogen permeation measurements

**Figure 6:**
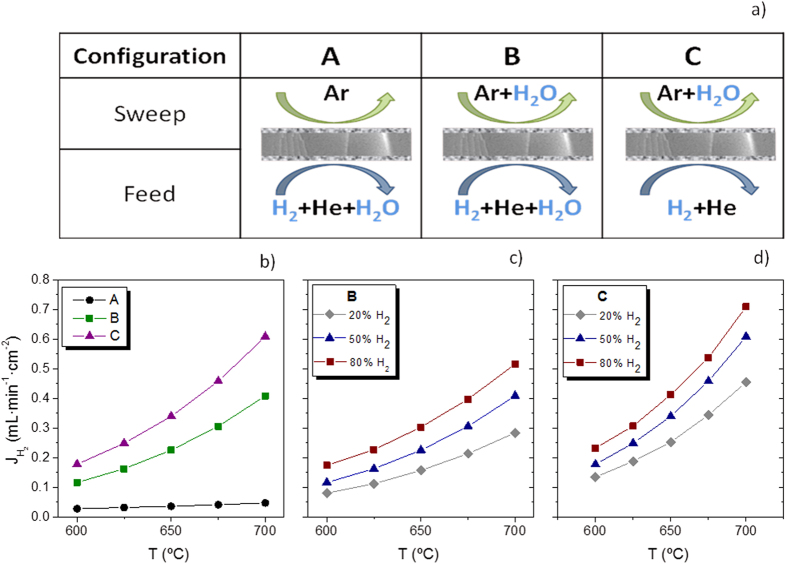
(**a**) Representation of configurations for membrane testing; (**b**) H_2_ flow as a function of temperature in the three configurations A, B and C with different hydration degree, feed stream 50% H_2_ in He; and (**c**,**d**) H_2_ flow as a function of temperature for different *p*H_2_ feeding in configuration B and C.

Permeation properties were thoroughly studied by analyzing the influence of the environment humidification and temperature on the H_2_ permeation. Three different configurations, depicted in Fig. 6a, were selected in order to study the effect of humidification: (A) only feed side humidified, (B) both membrane sides humidified and (C) only sweep side humidified. 50 vol.% H_2_ in He was employed as feed gas and Ar was used as sweep gas. Figure 6b plots the H_2_ permeation behavior through the composite membrane as a function of the temperature in the three configurations. When only the feed side is humidified (configuration A), H_2_ flow is below 0.05 mL·min^−1^·cm^−2^ due to the low concentration of protons as a result of the large *p*H_2_O gradient across the membrane. When both sides are humidified (configuration B), the obtained H_2_ flow is one order of magnitude higher than in configuration A (0.4 mL·min^−1^·cm^−2^ at 700 °C). This important increase is attributed to the faster proton transport through the membrane, as a consequence of the higher hydration of the material and hence the higher concentration of protonic defects^63^. In addition to proton transport, H_2_ is also generated at the sweep side by water splitting reaction mediated by the oxygen ion diffusion from the high *p*O_2_ side (Ar sweep) to the lower *p*O_2_ side (H_2_ feed). The oxygen transport takes place preferentially through the CYO phase, since this phase presents significant oxygen ion conductivity at elevated temperatures^80^, although it is still one order of magnitude lower than the electronic conductivity in reducing conditions. Finally, for humidified sweep gas (configuration C), the H_2_ flow increases further, reaching values of 0.61 mL·min^−1^·cm^−2^ at 700 °C. This increase in H_2_ production stems from the higher magnitude of water splitting process since a higher *p*O_2_ gradient is imposed across the membrane as compared to configuration B.

**Figure 7:**
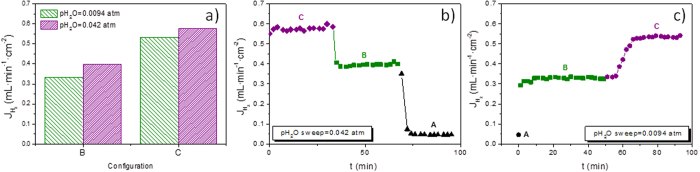
(**a**) H_2_ flows obtained at 700 °C in configurations B and C for *p*H_2_O(sweep) = 0.042 atm and *p*H_2_O(sweep) = 0.0094 atm. (**b**,**c**) H_2_ flow variation produced by the step-change from C to B to A with (**b**) *p*H_2_O (sweep) = 0.042 atm and (**c**) *p*H_2_O(sweep) = 0.0094 atm at 700 °C feeding 50 vol.% H_2_.

H_2_ permeation was also studied as a function of the hydrogen concentration (*p*H_2_) in the feed side (Fig. 6c,d). Irrespective of the humidification conditions, H_2_ flow rises with increasing *p*H_2_ as it is postulated by the Wagner equation, which describes the transport of both protons and oxygen ions. Furthermore, H_2_ permeation was investigated by using lower *p*H_2_O at the sweep side, *p*H_2_O = 0.0094 atm instead of 0.042 atm. Results are shown in Fig. 7a–c. H_2_ flows obtained at 700 °C sweeping *p*H_2_O = 0.042 atm are 19% and 8% higher than the values obtained for *p*H_2_O = 0.0094 atm, in configurations B and C, respectively (Fig. 7a). Assuming that the partial conductivities are constant in the studied *p*O_2_ and *p*H_2_O ranges, and that H_2_ transport can be described by Wagner’s, the H_2_ flow could be expressed as it is indicated in eq. 1, where the first term corresponds to the H_2_ permeating through the membrane and the second one responds to the H_2_ produced by water splitting.


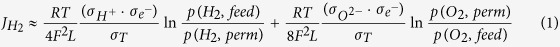


With *p*H_2_O = 0.042 atm, 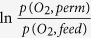 is 1.285 and 1.121 times higher than the corresponding to *p*H_2_O = 0.0094 atm in configuration B and C, respectively. On the other hand, the variation in the term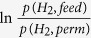 by changing the *p*H_2_O is almost negligible. From these results, it could be concluded that an important contribution to the H_2_ flow observed comes from the water splitting and the changes observed when *p*H_2_O decreases are mainly ascribed to the reduction of the oxygen transport through the membrane.

Step-changes from configurations C to A were monitored for both conditions (Fig. 7b,c). Hydrogen fluxes decrease changing from configuration C to B and A for *p*H_2_O = 0.042 atm (Fig. 7b) and they increase changing from configuration A to B and C for *p*H_2_O = 0.0094 atm (Fig. 7c). The quick response for both conditions is mainly related to the important changes in H_2_ production by water splitting^11^.

In the light of the previous reports on similar cercer composites^63^,^64^,^85^, the effect of Pt layer applied on the surface of the tested membrane on water splitting reaction must be also taken into consideration. According to^62^, no contribution of water splitting to the H_2_ permeation could be evidenced at lower water vapor pressure at the sweep side due to omitting Pt catalyst layer. However, cercer composites were just recently identified as promising candidates for H_2_ permeation membranes, therefore detailed studies have not been yet done to assign different reaction contributions to the overall H_2_ flux.

### Stability tests

**Figure 8:**
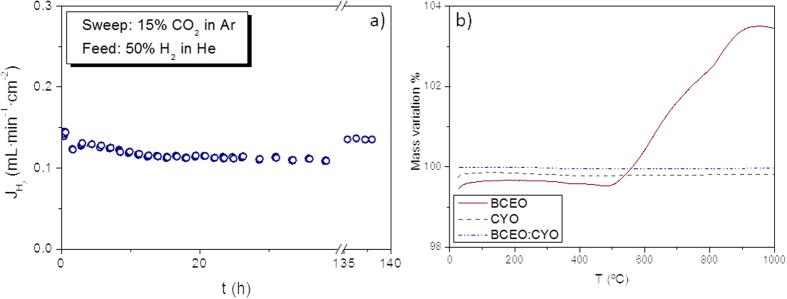
(**a**) H_2_ flow as a function of time by using 15 vol.% CO_2_ in Ar as sweep gas and 50 vol.% H_2_ in He as feed gas at 700 °C. Both sides of the membrane were humidified (configuration B). (**b**) TG measurements of BCEO, CYO and BCEO:CYO under 5 vol.% CO_2_ in Ar.

The assessment of the H_2_ permeation stability under CO_2_ atmospheres was carried out at 700 °C during 6 days by using 15 vol.% CO_2_ in Ar as sweep gas, 50 vol.% H_2_ in He as feed gas and humidifying both sides of the membrane (configuration B). Figure 8a shows that the H_2_ flow obtained under CO_2_ containing atmosphere is significantly lower than that obtained by using pure Ar, around 0.15 mL·min^−1^·cm^−2^. This drop in the H_2_ flux by using CO_2_ could be ascribed to the CO_2_/H_2_ and/or CO_2_/O_2_ competitive adsorption on the membrane surface^11^,^63^ that slows down the gas exchange. Besides, the flux equilibration in these conditions takes more than 2 days.

TG analysis was performed under 5 vol.% CO_2_ balanced with Ar by using crushed samples sintered at 1600 °C. Figure 8b indicates that CYO sample does not suffer any CO_2_ uptake under CO_2_ containing atmospheres within the studied temperature range. On the other hand, a mass gain is observed for BCEO sample above 500 °C, which is ascribed to the carbonation of the sample^37^,^66^,^73^. However, the mass of the composite BCEO:CYO does not increase indicating that no carbonation process is taking place. It can be concluded that the addition of ceria to the cerate allows shifting the carbonation equilibrium and consequently improves the stability of the composite.

**Figure 9:**
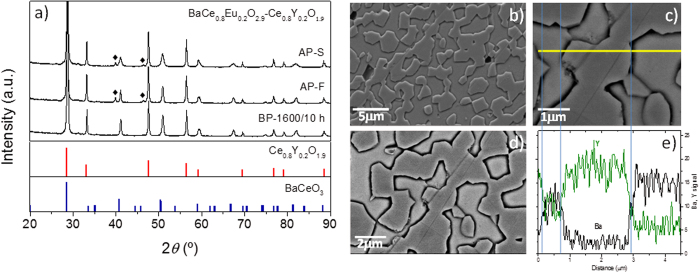
(**a**) Diffraction patterns for BCEO:CYO dual phase ceramic samples: as-sintered sample (BP-1600/10 h) and sample after H_2_-permeation tests (feed side AP-F and sweep sides AP-S). Peak positions corresponding to Pt traces from the catalytic layer are denoted by symbol (♦). As a reference (bottom): peak positions and their intensities for BaCeO_3_ and Ce_0.8_Y_0.2_O_1.9_. (**b**) SEM and (**c**,**d**) BSE-SEM micrographs and (**e**) EDS linescan analysis of the fractured cross-section of the BCEO:CYO membrane after the permeation measurements.

The structural stability of BCEO:CYO membrane after 13 days on stream (total duration of the permeation measurements, including CO_2_ stability measurement) was verified by XRD (Fig. 9a) and FE-SEM (Fig. 9b–e) analysis. Figure 9a depicts the XRD patterns recorded on dual-phase BCEO:CYO sintered samples before permeation (labeled BP-1600/10 h) and after the H_2_-permeation tests (labeled AP). Both the feed and sweep sides of the membrane were investigated to detect any phase changes or formation of carbonates (denoted with AP-F and AP-S for feed and sweep side of the membrane, respectively). As reference, the peak positions and related intensities for BaCeO_3_ and Ce_0.8_Y_0.2_O_1.9_ are presented at the bottom of the figure. Up to the analytical limits of the XRD device, no changes in the structure were found and both fluorite and perovskite phases are well distinguished. The peaks marked by a diamond correspond to Pt traces from the catalytic layer. The XRD pattern of the sweep side of the membrane after permeation shows broader peaks, which could indicate some modification in the surface microstructure.

Figure 9b–e presents the FE-SEM (b), and the BSE-SEM (c,d) micrographs and EDS linescan (e) analysis of the membrane after the permeation measurements (note that the membrane is the same specimen for all the H_2_ permeation measurements described in the work). Neither morphological nor structural changes were detected.

Finally, stability of dual phase composite material was checked after running the sintered membranes in continuous electrical tests for 670 h under 4% H_2_-containing dry reducing conditions, including several cycling steps from room temperature up to 900 °C and 1000 °C with holding times at these temperatures of 48 h and 20 h, respectively. TEM investigation of a reference sample and of post-treatment specimens is shown in Figure S4 in Supplementary Information. No morphological differences between the reference and post-treatment membranes and no grain boundary segregations were detected resulting from the 670 h continuous operation under reducing conditions.

## Conclusions

We have presented a dual phase material based on perovskite and fluorite exhibiting both high electronic and H^+^/O^−2^ co-ionic conductivity. Nominal BaCe_0.8_Eu_0.2_O_3-δ_ (BCEO) and Ce_0.8_Y_0.2_O_2-δ_ (CYO) were mixed in a 50:50 vol.% ratio. Eu^3+^ cations originally present in BCEO diffused to CYO during the sintering step (Ce_0.695_Y_0.2_Eu_0.105_O_2-δ_: CYEuO) as revealed by TEM analysis. DC-conductivity using a deuterium tracer showed that the composite is mixed protonic-electronic conductor (MPEC). Indeed, BCEO behaves as a prevailing proton conductor and CYO (CYEuO) as a mixed oxygen ionic and electronic conductor.

H_2_ permeation through a thick planar BCEO:CYO membrane was measured in different gas hydration configuration. The reached H_2_ fluxes are among the highest reported for ceramic membranes up to date 0.4 and 0.61 mL·min^−1^·cm^−2^ at 700 °C under configuration B and C, respectively (or thickness-normalized values of 0.02 and 0.0305 mL·min^−1^·cm^−1^ at 700 °C under configuration B and C, respectively). This remarkably high permeability originates from the transport of protons as well as from the hydrogen generated by water splitting on the permeate side (the high oxygen partial pressure side) of the membrane due to the oxygen ion transport in ceria. The membrane material was stable after 670 h continuous operation under 4% H_2_-containing dry reducing conditions, as well as in 15% CO_2_ containing atmosphere, although the resulting flux was lower than in pure Ar due to the competitive adsorption in the surface between H_2_/O_2_ and CO_2_. The stability in CO_2_ is tentatively attributed to the protective effect of the ceria phase over the cerate phase.

## Experimental Section

### Sample preparation

Materials with nominal composition BaCe_0.8_Eu_0.2_O_3-δ_ (BCEO) and Ce_0.8_Y_0.2_O_2-δ_ (CYO) were synthesized via the conventional solid**-**state route. Stoichiometric amounts of high**-**purity BaCO_3_ and CeO_2_, Eu_2_O_3_, Y_2_O_3_ (Sigma Aldrich) were weighted, mixed and ball-milled in ethanol. The solid**-**state synthesis for both compounds took place at 1400 °C for 6 h in air. A second calcination step (20 h) was required for the complete formation of the perovskite single phase, and resulting products were milled in ethanol. After drying, the two powder products were mixed in 50:50 vol.% ratio and were homogenized 24 h in ethanol, dried and sieved. Uniaxially pressed samples were sintered at 1600 °C for 10 h leading to formation of dual phase BCEO:CYO material with nominal stoichiometry BaCe_0.8_Eu_0.2_O_3-δ_:Ce_0.8_Y_0.2_O_2-δ_. Samples were grinded to remove the defective top layer and no Ba-deficiency or secondary oxide exclusions compensating eventual Ba evaporation were detected by ICP-OES. Relative density of more than 98% was determined via the Archimedes approach on sintered dual phase composite samples.

### Characterization techniques

Inductively coupled plasma optical emission spectrometry (ICP-OES) analysis was used to monitor chemical composition of the dual phase ceramics by quantification of the cation content. 50 mg sample powder was mixed with 2 mL HClO_4_ (NORMATOM^®^, VWR Chemicals, Germany) and heated for 30 min to fuming. After cooling, 1 mL H_2_O_2_ (NORMAPUR) and 1 mL HCl (Merck Suprapur, Germany) were added. Samples were heated at 70 °C for 30 min until complete dissolution of solid content and finally 50 mL volumes were prepared. Samples were measured at dilution 1:20 with a Thermo Scientific iCAP 7600 dual-view spectrometer, each sample was measured twice and the mean result of three emission lines per element was used for quantification. External calibration was performed with standards prepared by dilution of Merck Certipur^®^ certified plasma emission standards with diluted acids. Relative standard deviation is 1–3%.

Powder X-ray diffraction (XRD) patterns of BCEO, CYO and composite BCEO:CYO as prepared and sintered were recorded in the 2 theta range from 10° to 80° using D4 ENDEAVOR diffractometer by Bruker AXS with CuKα radiation (λ = 1.54 Å). XRD patterns of BCEO:CYO membrane after permeation tests were recorded in the 2 theta range from 20° to 90° using PANalytical Cubix fast diffractometer with CuKα_1,2_ radiation and an X’Celerator detector in Bragg-Brentano geometry. Phase identification was carried out with ICDD PDF2**-**Database (Release 2004) and X′Pert Highscore Plus (by PANalytical). The TOPAS V4 software (Bruker AXS) was used to determine the lattice parameters and for Rietveld refinements. The stoichiometry of each phase is fixed for the Rietveld refinement and the starting crystal structure model was obtained from the Inorganic Crystal Structure Database (ICSD). The final weighted R-factor (Rwp, %) for each refinement is listed in Table 2.

Microstructural and chemical analyses of BCEO and CYO, as well as of the dual phase samples were performed by means of field emission scanning electron microscopy (FE-SEM) (Zeiss Ultra 55) equipped with energy-dispersive X-ray spectroscopy (EDS) (INCA, Oxford). Transmission electron microscopy (TEM) analysis was carried out using a Tecnai TF 20 UT equipped with EDS and a Gatan’s imaging filter (GIF) operated at 200 kV. High-resolution scanning TEM high angle annular dark field (STEM-HAADF) imaging was performed by using C_s_ probe corrected FEI-Titan 80–300 STEM instrument operating at 300 kV. Electron-transparent specimens were prepared from as-sintered pellets using the traditional approach which includes ultrasonic drilling, mechanical polishing and Ar^+^ ion milling at 5 keV.

Thermal expansion coefficient (TEC) of the dual phase ceramics was measured on sintered bar samples with dimensions 25 × 4 × 4 mm^3^ from 30 to 1400 °C (heating rate 3 °C/min) in air using Netzsch Dil 402C. Linear thermal expansion coefficients α under heating and cooling were calculated using Eq. (2), where L_0_ and dL are respectively the initial length and the length change of the sample, while T_0_ and T are the starting and the final temperature, respectively:


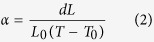


Electrical conductivity measurements were carried out by the standard four-point DC technique on sintered bars. Rectangular bar specimens were prepared from the resulting powders through uniaxial pressing at 100 MPa and subsequently sintered at 1600 °C. Silver paste and wire were used for contacting. The constant current was supplied by a programmable current source (Keithley 2601), while the voltage drop through the sample was detected by a multimeter (Keithley 3706). The voltage was measured by the current in both forward and reverse directions, in order to eliminate the thermal effect and to avoid non-ohmic responses. H/D isotopic effect and hydration effect for the separate materials and the dual phase material BCEO:CYO, were studied under reducing conditions: 5 vol.% H_2_ in He and 5 vol.% D_2_ in He both dry and moist (H_2_ and D_2_ humidified with H_2_O and D_2_O at room temperature, respectively). The influence of the *pO*_*2*_ on total conductivity was also evaluated under wet reducing atmospheres by using: wet 0.05 vol.%, 5 vol.% and 100 vol.% H_2_ and wet 5 vol.% D_2_.

Temperature-programmed reduction (TPR) was performed using 2910 Micromeritics equipment on crushed and ground samples sintered at 1600 °C. Powders were degassed under a dry Ar flow for 1 h and then were subjected to reduction under a dry H_2_/Ar (1/9) flow, and a heating rate of 10 °C·min^−1^ until 900 °C. H_2_ consumption was measured by a TCD (thermal conductivity detector).

H_2_ permeation measurements were performed in a multistep continuous process using a BCEO:CYO dense disk with a diameter of 15 mm and thickness of 500 μm sintered at 1600 °C. Both disk surfaces were coated with a 20 μm screen printed Pt porous layer aiming to improve the surface catalytic activity and evaluate predominantly the bulk H_2_ transport. Permeation measurements were performed on a sealed sample in double chamber quartz reactor following the methodology reported elsewhere^25^,^86^. Three different hydration conditions were evaluated: (A) feed side humidified and sweep side dry; (B) both sides humidified; (C) only sweep side humidified, where humidification means *p*H_2_O = 0.042 atm and dry corresponds to *p*H_2_O = 2·10^−5^ atm (bottle dry). Ar was used as sweep gas and a 50 vol.% H_2_ in He mixture was employed as feed (1.18 atm absolute pressure) under all the mentioned conditions. The flow rates used were 100 mL·min^−1^ for feed and 150 mL·min^−1^ for sweep and they were controlled using mass flow controllers (MFCs). The hydrogen content in the permeate side was analyzed using micro-GC Varian CP-4900 equipped with Molsieve5A and PoraPlot-Q glass capillary modules. Sealing was obtained using a silver ring and applying a spring load. Sealing was confirmed by continuous monitoring the He concentration in the permeate stream and it was considered adequate when the helium concentration was lower than 5% of the H_2_ permeated.

In order to evaluate the stability in operation of the composite under CO_2_ containing atmospheres, H_2_ permeation measurements were also performed at 700 °C for 6 days by using 15 vol.% CO_2_ in Ar as sweep gas. After permeation measurements, integrity of the sample was evaluated by means of XRD and SEM analysis. Scheme of the steps of the hydrogen permeation measurements in a continuous process is given in Supplementary information, Figure S1.

Additionally, TG measurements were carried out on the BCEO, CYO and the composite BCEO:CYO by using crushed samples sintered at 1600 °C. TG was performed in a flow of dry 5% CO_2_ in Ar from 25 to 1000 °C with a heating ramp of 10 K·min^−1^ by using a Mettler-Toledo StarE balance.

## Additional Information

**How to cite this article**: Ivanova, M. E. *et al*. Hydrogen separation through tailored dual phase membranes with nominal composition BaCe_0.8_Eu_0.2_O_3-δ_:Ce_0.8_Y_0.2_O_2-δ_ at intermediate temperatures. *Sci. Rep.* **6**, 34773; doi: 10.1038/srep34773 (2016).

**Publisher’s note:** Springer Nature remains neutral with regard to jurisdictional claims in published maps and institutional affiliations.

## Supplementary Material

Supplementary Information

## Figures and Tables

**Table 1:** Based on literature summary of thickness (*L*_*mem*_)-normalized values of H_2_ permeation rates *j*_*H2, norm*_ through ceramic membranes.

Material	Ref.	Gas atmosphere Feed – Sweep	T (°C)	*j*_*H2, norm*_(mL·min^−1^·cm^−1^)
*Defective fuorite structured materials*				
La_5.5_WO_11.25-δ_	25	wet 50% H_2_ in He – wet Ar	750/900	1.5·10^−3^/4.7·10^−3^
La_5.5_W_0.8_Mo_0.2_O_11.25-δ_	25	wet 50% H_2_ in He – wet Ar	700	2.7·10^−3^
La_5.5_W_0.8_Re_0.2_O_11.25-δ_	25	wet 50% H_2_ in He – wet Ar	700	5.9·10^−3^
Nd_5.5_WO_11.25-δ_	34	wet 20% H_2_ in He – wet Ar	1000	1.2·10^−3^
(Nd_5/6_La_1/6_)_5.5_WO_12-δ_	35	wet 50% H_2_ in He – wet Ar	900	1.2·10^−3^
(La_5/6_Nd_1/6_)_5.5_WO_12-δ_	36	wet 50% H_2_ in He – wet Ar	900	1.4·10^−3^
Nd_5.5_W_0.5_Mo_0.5_O_11.25-δ_	37	wet 50% H_2_ in He – wet Ar	900	7.5·10^−3^
Nd_5.5_W_0.5_Re_0.5_O_11.25-δ_	38	wet 50% H_2_ in He – wet Ar	900	4.1·10^−3^
*Perovskite structured materials*				
BaCe_0.80_Y_0.10_Ru_0.10_O_3-δ_	39	wet H_2_ in Ar – Ar	800	4.3·10^−3^
BaCe_0.95_Nd_0.05_O_3-δ_	40	wet 80% H_2_ in He – dry Ar + Ne	900	1.3·10^−3^
BaZr_0.80_Y_0.15_Mn_0.05_O_3-δ_	41	wet 50% H_2_ in He – wet Ar	900	1.4·10^−3^
SrCe_0.95_Tm_0.05_O_3-δ_	42	10% H_2_ in He – air	700/900	1.2·10^−3^/1.9·10^−3^
SrCe_0.95_Tm_0.05_O_3-δ_	43	10% H_2_ in He – air	700/900	2.6·10^−3^/6.4·10^−3^
SrCe_0.95_Tm_0.05_O_3-δ_	44	10% H_2_ dry in He – 20% O_2_ in Ar	750/900	4.3·10^−3^/6.8·10^−3^
SrCe_0.95_Tb_0.05_O_3-δ_	45	20% H_2_ in He – CO in Ar	750/900	4.0·10^−4^/1.6·10^−3^
SrCe_0.95_Y_0.05_O_3-δ_	46	80% H_2_ in He – Ar	900	3.2·10^−4^
SrCe_0.95_Yb_0.05_O_3-δ_	47	10% H_2_ in He – air	677	2.7·10^−3^
SrCe_0.95_Eu_0.05_O_3-δ_	48	100% H_2_ – He	700/850	2.3·10^−4^/7.4·10^−4^
SrCe_0.95_Sm_0.05_O_3-δ_	48	100% H_2_ – He	850	5.4·10^−4^
SrCe_0.75_Zr_0.20_Tm_0.05_O_3-δ_	49	H_2_ in He – wet Ar	900	5.0·10^−3^
SrCe_0.75_Zr_0.20_Tm_0.05_O_3-δ_	44	10% H_2_ dry in He – 20% O_2_ in Ar	750/900	8.0·10^−4^/2.4·10^−3^
SrCe_0.70_Zr_0.25_Ln_0.05_O_3-δ_ (Ln = Tm, Yb)	50	wet 20% H_2_ – wet sweep (not specified)	900	2.3·10^−4^
SrCe_0.65_Zr_0.20_Eu_0.15_O_3-δ_	51	100% H_2_ – He	900	8.5·10^−4^
SrZr_0.95_Y_0.05_O_3-δ_	47	20% H_2_ – air in He	700	<2.3·10^−5^
*Cer-Met dual phase materials*				
BaCe_0.95_Tb_0.05_O_3-δ_:Ni (50:50 wt.%)	52	50% H_2_ in N_2_ – He	850	8.3·10^−3^
BaCe_0.90_Y_0.10_O_3-δ_:Ni (60:40 vol.%)	53	4% H_2_ in He – 100 ppm H_2_ in N_2_	800	1.7·10^−2^
BaCe_0.80_Y_0.20_O_3-δ_:Ni (60:40 vol.%)	54	3.8% H_2_ in N_2_ – 100 ppm H_2_ in N_2_	900	2.0·10^−3^
BaCe_0.85_Zr_0.10_Tb_0.05_O_3-δ_:Ni (50:50 wt.%)	55	50% H_2_ in 50% He -Ar	800	8.5·10^−3^
BaCe_0.70_Zr_0.10_Y_0.20_O_3-δ_:Ni (60:40 vol.%)	56	4% H_2_ in He – 100 ppm H_2_ in N_2_	900	5.6·10^−3^
BaCe_0.70_Zr_0.10_Y_0.10_Yb_0.10_O_3-δ_:Ni (60:40 vol.%)	57	20% H_2 _wet, 60% CO_2_, 20% He - N_2_	900	3.5·10^−3^
Ce_0.50_La_0.4875_Ca_0.0125_O_2-δ_:Ni (60:40 vol.%)	58	wet 20% H_2_, 77% N_2_ – Ar	900	1.5·10^−3^
YSZ:Pd (40:60 vol.%)	59	90% H_2_ in He – N_2_	400/900	4.7·10^−2^/9.4·10^−2^
*Cer-Cer dual phase materials*				
La_5.5_WO_11.25-δ_:La_0.87_Sr_0.13_CrO_3-δ_ (50:50 vol.%)	60	wet 50% H_2_ in He – wet Ar	700	5.5·10^−3^
BaCe_0.80_Eu_0.20_O_3-δ_:Ce_0.80_Eu_0.20_O_2-δ_ (50:50 vol.%)	61	H_2_, CH_4_, H_2_O, CO, CO_2_ – He	900	<7.0·10^−2^
BaCe_0.80_Y_0.20_O_3-δ_:Ce_0.80_Y_0.20_O_2-δ_ (50:50 wt.%)	62	wet 50% H_2_ in He – wet Ar	900	1.1·10^−2^
BaCe_0.65_Zr_0.20_Y_0.15_O_3-δ_:Ce_0.85_Gd_0.15_O_2-δ_ (50:50 vol.%)	63	wet 50% H_2_ in He – wet Ar	755	1.76·10^−2^
BaCe_0.20_Zr_0.70_Y_0.10_O_3-δ_:Sr_0.95_Ti_0.90_Ni_0.10_O_3-δ_ (50:50 vol.%)	64	9% H_2_ in He – dry Ar	800	1.1·10^−3^
SrZrO_3_:SrFeO_3_ (80:20 vol.%)	65	H_2_ in He – wet Ar	900	4.8·10^−3^

**Table 2:** Rietveld refinement results from the XRD patterns of BCEO and CYO synthesized via the conventional solid-state route at 1400 °C during 6 h and 20 h (in total 26 h), and of BCEO:CYO dual-phase pellet (50:50 vol.%) sintered at 1600 °C for 10 h. For reference, the lattice parameters of pure BaCeO_3_ are a = 8.776(1) Å, b = 6.234(1) Å, c = 6.214(1) Å.

Nominal starting compounds	Phase composition	Space group	Lattice parameter(s), (Å)	Phase wt. %	Phase D_th_ (g/cm^3^)	Rwp, %
BaCe_0.8_Eu_0.2_O_2.9_ 1400 °C/6 h	BaCe_0.8_Eu_0.2_O_2.9_ tetragonal	P-42m	a = 4.388 (1) c = 4.465 (1)	86	6.30	6.7
	BaCe_0.8_Eu_0.2_O_2.9_ orthorhombic	Pmcn	a = 6.245 (1) b = 8.770 (1) c = 6.272 (1)	14	6.31	
BaCe_0.8_Eu_0.2_O_2.9_ 1400 °C/26 h	BaCe_0.8_Eu_0.2_O_2.9_ orthorhombic	Pmcn	a = 6.229 b = 8.782 c = 6.257 (1)	100	6.35	6.6
Ce_0.8_Y_0.2_O_1.9_ 1400 °C/6 h	Ce_0.9_Y_0.1_O_1.95_ cubic	Fm-3m	a = 5.411(1)	45	6.93	5.3
	Ce_0.8_Y_0.2_O_1.9_ cubic	Fm-3m	a = 5.401(1)	55	6.75	
Ce_0.8_Y_0.2_O_1.9_ 1400 °C/26 h	Ce_0.8_Y_0.2_O_1.9_ cubic	Fm-3m	a = 5.405(1)	100	6.8	5.9
BaCe_0.8_Eu_0.2_O_2.9_: Ce_0.8_Y_0.2_O_1.9_ 1600 °C/10 h	BaCe_1-x_Eu_x_O_3-δ_ orthorhombic	Pmcn	a = 8.786(1) b = 6.245(1) c = 6.210(1)	46	6.34	5.4
	Ce_0.695_Y_0.2_Eu_0.105_O_2-δ_ cubic	Fm-3m	a = 5.411(1)	54	6.77	
