# Supplementary material for: Hydrogen separation through tailored dual phase membranes with nominal composition BaCe0.8Eu0.2O3-δ:Ce0.8Y0.2O2-δ at intermediate temperatures
Source: Sci Rep. 2016 Nov 4;6:34773. doi: 10.1038/srep34773 (PMC5095711; doi:10.1038/srep34773)
Supplement: Supplementary Information [file srep34773-s1.pdf]

Hydrogen separation through tailored dual phase membranes with nominal composition $BaCe_{0.8}Eu_{0.2}O_{3-\delta}$:$Ce_{0.8}Y_{0.2}O_{2-\delta}$ at intermediate temperatures

Mariya E. Ivanova*[1], Sonia Escolástico[2], Maria Balaguer[1], Justinas Palisaitis[3], Yoo Jung Sohn[1], Wilhelm A. Meulenberg[1], Olivier Guillon[1], Joachim Mayer[3], Jose M. Serra*[2]

[1] *Institute of Energy and Climate Research IEK-1, Forschungszentrum Jülich GmbH, D-52425 Jülich, Germany*

[2] *Instituto de Tecnología Química, Universidad Politécnica de Valencia-Consejo Superior de Investigaciones Científicas, Av. Naranjos s/n, E-46022 Valencia, Spain*

[3] *Ernst Ruska-Centre for Microscopy and Spectroscopy with Electrons ER-C, Forschungszentrum Jülich GmbH, D-52425 Jülich and Central Facility for Electron Microscopy GFE, RWTH Aachen University, 52074 Aachen, Germany*

Figure S1

Figure S1. Scheme of the hydrogen permeation measurements showing all steps in one continuous process. At the end of this complex process, material remained stable and membrane preserved its integrity.

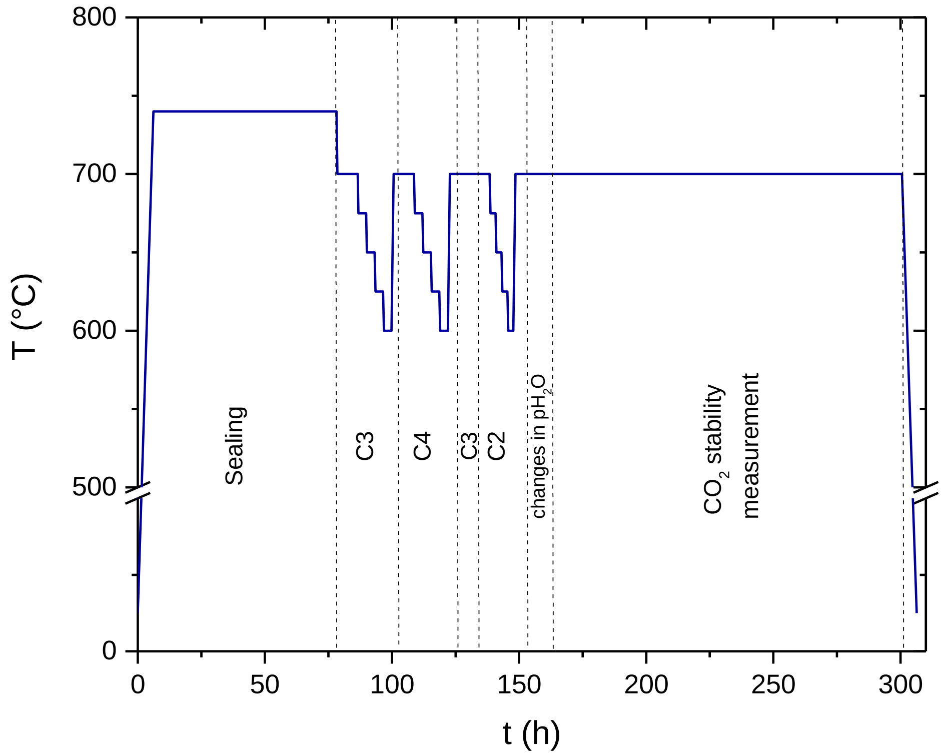

Figure S2

Figure S2. Thermal expansion coefficients of ceramic composite BCEO:CYO as a function of the temperature. Linear TEC (heating from 30 to 1400 °C): $12.4 \cdot 10^{-6}$ K^{-1}, linear TEC (cooling from 1400 to 60 °C): $12.2 \cdot 10^{-6}$ K^{-1}

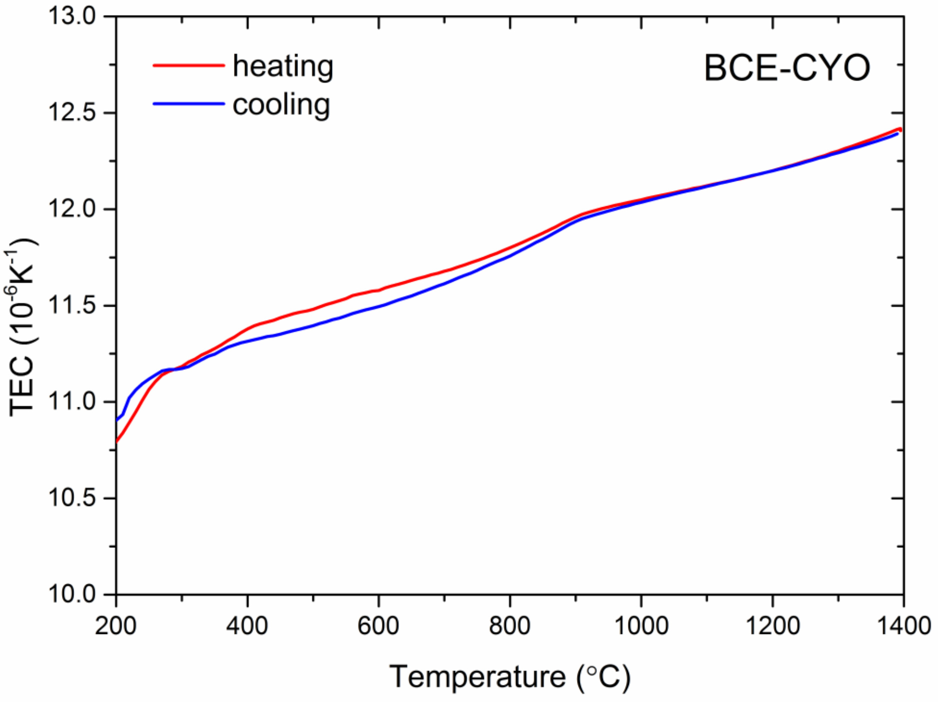

Figure S3

Figure S3. TPR evolution in dry H_2 (10% in Ar) for CYO and BCEO single phase compounds, and for BCEO:CYO dual phase composite as crushed and grinded membranes sintered at 1600 ºC (heating ramp 10 ºC·min^{-1})

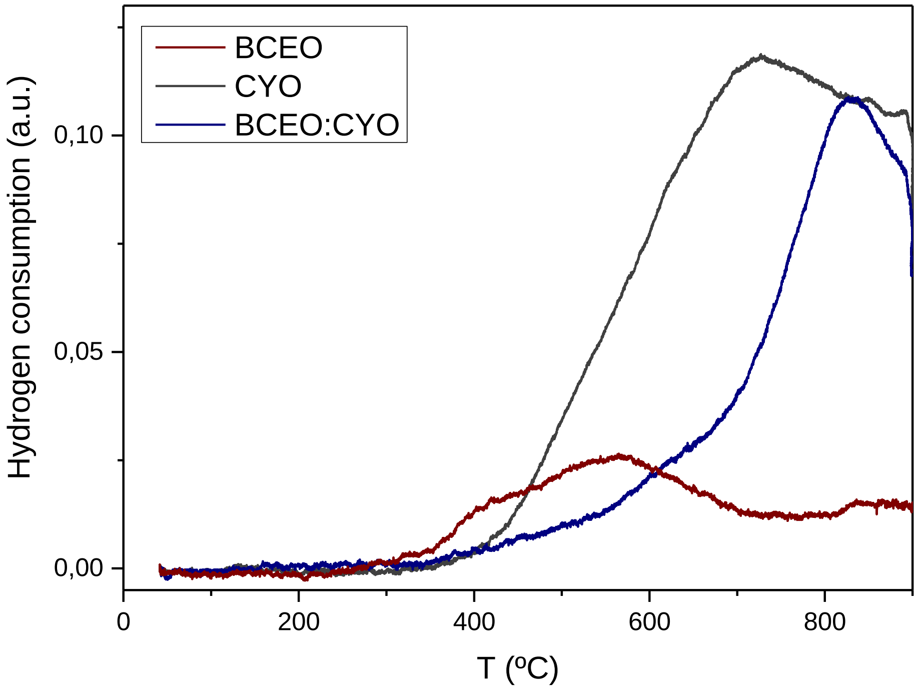

Figure S4

Figure S4. TEM images of $BaCe_{1-x}Eu_xO_{3-\delta}$:$Ce_{1-y}(Y,Eu)_yO_{2-\delta}$: a) reference sample sintered at 1600 °C/10h; b) and c) post-treatment sample subjected to continuous electrical measurements for 670 h conducted in 4% H_2-containing dry conditions with cycles from RT to 900 °C/48h and 1000 °C/24h.

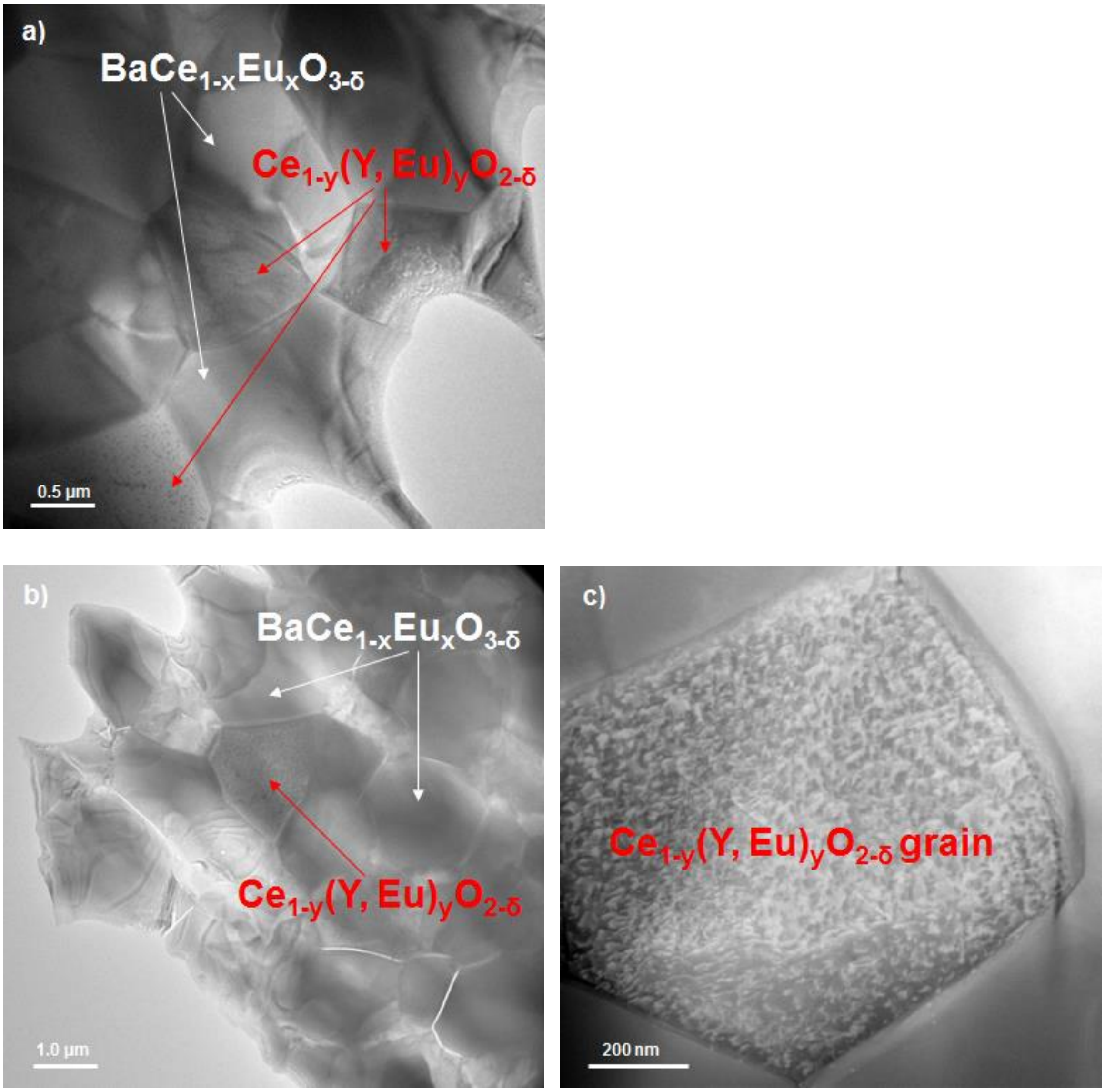

Figure S5

Figure S5. a) SEM mapping images of $BaCe_{1-x}Eu_xO_{3-\delta}$:$Ce_{1-y}(Y,Eu)_yO_{2-\delta}$ sample sintered at 1600 °C/10h and b) EDX spectra of (nominally) CYO and BCEO grains.

It is observed that the Eu (yellow) is preferably distributed along with the Y (pink). The EDX spectra shows also that the Eu is in the fluorite grains.

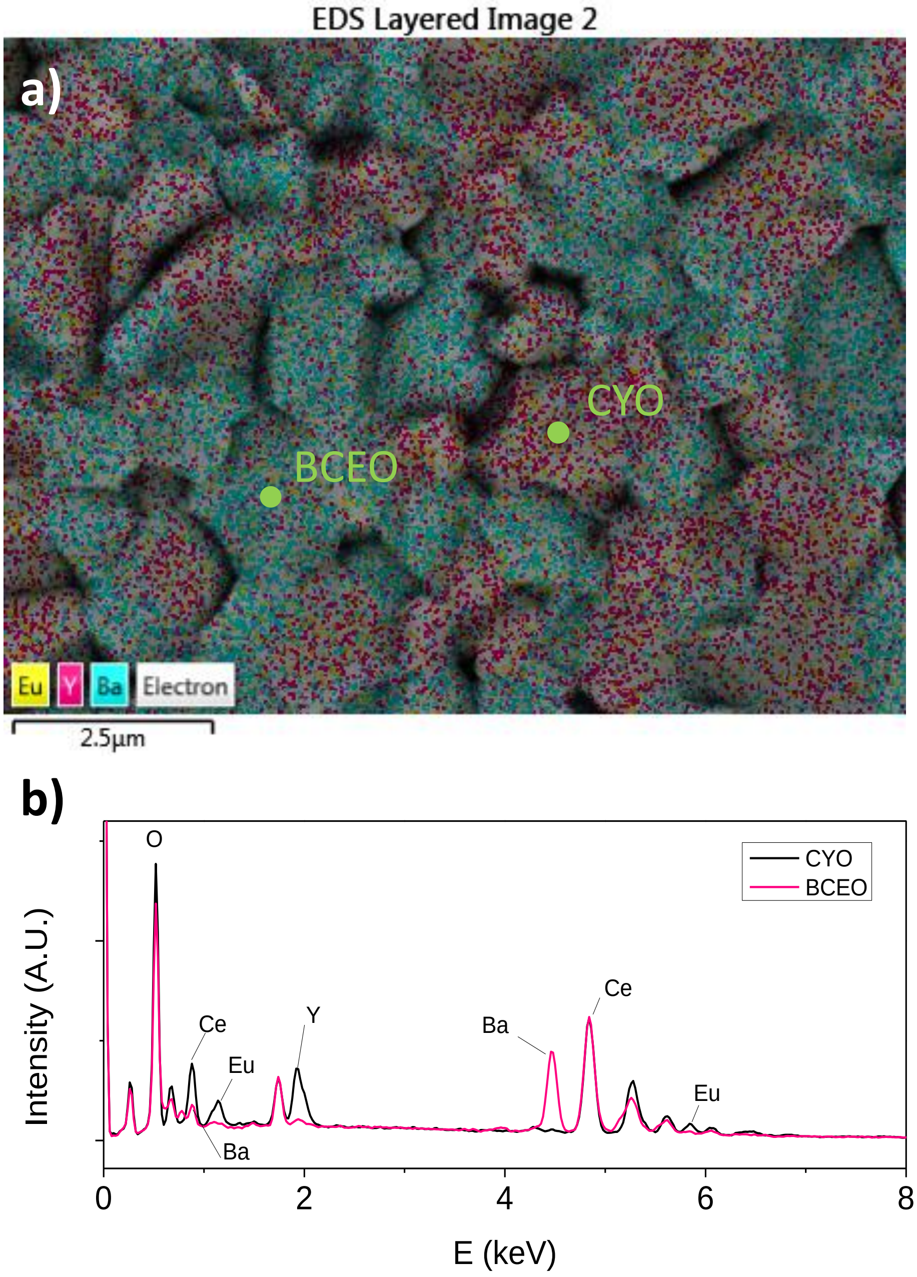

Table S1

Table S1. ICP-OES results in percentage of mass (AW-%) and standard deviations (SD-%) for the single phases CYO and BCEO, and for the composite mixed (no thermal treatment) and sintered at 1600 ºC. The stoichiometry (in mole) is calculated from ICP-OES. For the composite phases the following assumptions have been done: (i) the Eu in the mixed BCEO-CYO is in the perovskite phase (P), since no thermal treatment was carried out; (ii) the Eu in the sintered BCEO-CYO is in the fluorite phase (F), based on FESEM and TEM results that do not detect Eu in the perovskite. The mass balance indicates that the barium was not deficient after the sintering.

Compound	Phase	Ba			Ce			Y			Eu		
		AW (%)	SD (%)	mole	AW (%)	SD (%)	mole	AW (%)	SD (%)	mole	AW (%)	SD (%)	mole
**CYO**	*F*	<0,001		**0**	68,2	0,6	**0.797**	11,0	0,1	**0.203**	<0,0005		**0**
**BCEO**	*P*	39,6	0,7	**1.005**	32,2	0,7	**0.801**			**0**	8,5	0,2	**0,195**
**BCEO:CYO mixed**	*F*	18,4	0,2	**0**	48	0,4	**0.802**	5,32	0,05	**0.198**	3,63	0,04	**0**
	P			**1.04**			**0.778**			**0**			**0,185**
**BCEO:CYO sintered**	*F*	19,4	0,1	**0**	53	0,1	**0.619**	5,97	0,03	**0.203**	3,84	0,02	**0,09**
	P			**1.005**			**0.949**			**0**			**0**
